# Peripheral nerve‐derived fibroblasts promote neurite outgrowth in adult dorsal root ganglion neurons more effectively than skin‐derived fibroblasts

**DOI:** 10.1113/EP090751

**Published:** 2023-02-28

**Authors:** Masato Hara, Ken Kadoya, Takeshi Endo, Norimasa Iwasaki

**Affiliations:** ^1^ Department of Orthopaedic Surgery, Faculty of Medicine and Graduate School of Medicine Hokkaido University Sapporo Japan

**Keywords:** fibroblast, peripheral nerve, regeneration

## Abstract

Although fibroblasts (Fb) are components of a peripheral nerve involved in the regenerative process associated with peripheral nerve injury, detailed information regarding their characteristics is largely lacking. The objective of the present study was to investigate the capacity of Fb derived from peripheral nerves to stimulate the outgrowth of neurites from adult dorsal root ganglion neurons and to clarify their molecular characteristics. Fibroblasts were prepared from the epineurium and parenchyma of rat sciatic nerves and skin. The Fb derived from epineurium showed the greatest effect on neurite outgrowth, followed by the Fb derived from parenchyma, indicating that Fb derived from nerves promote neurite outgrowth more effectively than skin‐derived Fb. Although both soluble and cell‐surface factors contributed evenly to the neurite‐promoting effect of nerve‐derived Fb, in crush and transection injury models, Fb were not closely associated with regenerating axons, indicating that only soluble factors from Fb are available to regenerating axons. A transcriptome analysis revealed that the molecular profiles of these Fb were distinctly different and that the gene expression profiles of soluble factors that promote axonal growth are unique to each Fb. These findings indicate that Fb are molecularly and functionally different depending on their localization in nerve tissue and that Fb derived from epineurium might be involved more than was previously thought in axon regeneration after peripheral nerve injury.

## Abstract (highlights)

- **What is the central question of this study?**
  Although fibroblasts are involved in the regenerative process associated with peripheral nerve injury, detailed information regarding their characteristics is largely lacking.
- **What is the main finding and its importance?**
  Nerve‐derived fibroblasts have a greater neurite‐promoting effect than skin‐derived fibroblasts, and epineurium‐derived fibroblasts can promote neurite outgrowth more effectively than parenchyma‐derived fibroblasts. The epineurium‐derived fibroblasts and parenchyma‐derived fibroblasts have distinctly different molecular profiles, including genes of soluble factors to promote axonal growth. Fibroblasts are molecularly and functionally different depending on their localization in nerve tissue, and epineurium‐derived fibroblasts might be involved in axon regeneration after peripheral nerve injury more than previously thought.

## 1 INTRODUCTION

Although it is possible for the peripheral nervous system (PNS) to regenerate, clinical results after peripheral nerve injury (PNI) are not always satisfactory (Evans et al., 1994; Lundborg, 2000), which indicates the need for development of an effective therapy for the treatment of PNI (Min et al., 2021). However, the detailed mechanisms responsible for the regenerative process after PNI remain to be elucidated fully. An accumulated body of evidence suggests that regeneration after PNI is induced by a series of orchestrated interactions in a variety of cells, which include Schwann cells (SCs), macrophages, vascular endothelial cells (ECs) and fibroblasts (Fb).

Schwann cells differentiate into a reparative phenotype referred to as repair SCs that function to remove myelin debris, provide aid in the survival of damaged neurons, promote axon regeneration and remyelinate newly formed axons (Jessen & Arthur‐Farraj, 2019; Napoli et al., 2012). Macrophages contribute to the formation of the initial tissue that bridges the site of a nerve transection, to guide growing axons for their precise targeting at the site of the lesion (Dun et al., 2019; Li et al., 2022), to remove myelin debris and to promote axon regeneration in the region of Wallerian degeneration (Boissonnas et al., 2020). Vascular ECs guide the migrating SCs to the lesion site (Cattin et al., 2015), and in the region of Wallerian degeneration they promote macrophage accumulation and supply the neurotrophic factors required for regeneration (Caillaud et al., 2019).

Regarding Fb, it has been shown that they guide the directional migration of SCs at the lesion site (Parrinello et al., 2010), but the issue of whether Fb contribute directly to axon regeneration remains to be determined. Fibroblasts are generally thought to be indispensable for tissue repair because of their great proliferative capacity to fill tissue defects in various organs with an extracellular matrix (Harris et al., 2017). Recent progress has clarified that tissue‐specific Fb have distinct roles in maintaining steady‐state conditions, in causing disease conditions to develop and in promoting tissue repair (Plikus et al., 2021). For example, in the steady state, Fb in the renal interstitium produce erythropoietin to maintain the production of red blood cells and to reduce their production after renal damage that can cause renal anaemia (Yamazaki et al., 2013). Hepatic satellite cells protect spared hepatic tissue after acute hepatic injury, while they exaggerate hepatic damage by promoting hepatic fibrosis in conditions of chronic inflammation (Bandyopadhyay et al., 2011). It is noteworthy that the functions of Fb differ depending on their precise anatomical locations, even in the same organ. For example, in the lung, peri‐alveolar Fb provide lipids to type 2 alveolar epithelial cells to produce surfactants (Barkauskas et al., 2013), whereas adventitial Fb in bronchovascular bundles promote lung fibrosis (Tsukui et al., 2020).

Given the above, it is reasonable to assume that Fb at different anatomical locations would have different functions and effects on the repair process, including axon regeneration after PNI. Therefore, we hypothesize that the capability of Fb to stimulate axon regeneration is dependent on the specific location of Fb in the PNS. The purpose of the present study was to investigate the capacity of Fb derived from peripheral nerve tissue to stimulate neurite outgrowth in adult dorsal root ganglion (DRG) neurons and to clarify their molecular characteristics.

## 2 METHODS

### 2.1 Ethical approval

The study protocol was approved by the local ethical committee of Hokkaido University (17‐0071). All procedures conformed to the principles and regulations of *Experimental Physiology* (Grundy, 2015; Percie du Sert et al., 2020).

### 2.2 Animals

Adult Lewis rats (wild‐type; Charles River Laboratories Japan; 10–20 weeks of age; males and females) were used in all experiments. The animals had free access to food and water throughout the study. For general anaesthesia, a mixture of ketamine (75–100 mg/kg; Ketalar; Daiichi Sankyo Propharma Corporation, Tokyo, Japan) and medetomidine (0.5 mg/kg; Domitor; Orion Corporation, Espoo, Finland) was administered by i.p. injection. For postoperative analgesia, buprenorphine (0.05 mg/kg; Buprenorphine Inj; NISSIN, Yamagata, Japan) was administered s.c. every 24 h starting immediately after surgery for a total of four doses up to 72 h. For cell preparations, rats were killed by cervical spine dislocation under deep anaesthesia by i.p. injection of ketamine (100 mg/kg).

### 2.3 Fibroblast preparation

Sciatic nerves were dissected from two male rats (10 weeks of age) immediately post mortem. The epineurium and parenchyma were separated manually from nerves with micro‐forceps. Abdominal skin (1 cm^2^) was dissected from a different male rat (10 weeks of age) post mortem, followed by the removal of subcutaneous adipose tissue. These tissues were cut into 1–2 mm pieces using micro‐scissors and transferred to enzymatic digestion medium containing 2% collagenase I (Sigma‐Aldrich, St Louis, MO, USA) and 0.125% trypsin in Dulbecco's modified Eagle's medium (DMEM)/Ham's F‐12 (Wako, Osaka, Japan). After enzymatic incubation for 1 h at 37°C, 10% fetal bovine serum (FBS; Sigma‐Aldrich) in DMEM was added to stop the enzymatic digestion. After centrifugation and removal of the supernatant, the pieces were dissociated mechanically by 30 cycles of pipetting in Fb culture medium consisting of DMEM/Ham's F‐12 with 10% FBS, 1% GlutaMAX (Thermo Fisher Scientific, Waltham, MA, USA) and 1% penicillin–streptomycin (P/S; Thermo Fisher Scientific). The cell suspension was then filtered through a 40 μm cell strainer (Greiner Bio‐One, Kremsmünster, Austria) to remove tissue pieces. The viability of the dissociated cells was assessed by Trypan Blue (Thermo Fisher Scientific) and was within the range of 92–96%. Dissociated cells were seeded onto a non‐coated 75 cm^2^ flask, followed by the replacement of the culture medium 30 min later. After a single passaging 3–4 days later, the cells were subjected to fixation with 4% paraformaldehyde (PFA), co‐culture with DRG neurons and RNA sequencevs analysis.

### 2.4 Dorsal root ganglion neuron preparation

Dorsal root ganglion neurons were prepared from two 10‐week‐old male rats as described previously (Endo, Kadoya, Kawamura et al., 2019). Each DRG neuron preparation used one rat. These animals were different from those used for Fb preparation. In brief, bilateral lumbar DRGs were dissected, cut into 1–2 mm pieces using micro‐scissors and incubated in an enzymatic digestion medium consisting of DMEM/Ham's F‐12 with 1% collagenase XI (Sigma‐Aldrich) for 1 h at 37°C. After adding DMEM with 10% FBS to stop the enzymatic reaction, the tissue pieces were resuspended in 1 ml of neuron culture medium consisting of DMEM/Ham's F‐12 supplemented with 2% B27 supplement (Thermo Fisher Scientific) and 1% P/S. The tissue pieces were gently triturated 30 times with a 1 ml pipette to give a single‐cell suspension of DRG neurons.

### 2.5 Co‐culture of Fb and DRG neurons

Two types of fibroblast–neuron co‐culture methods were used. In the first method, the neurons were cultured on top of the Fb. The three types of Fb [derived from epineurium (Fb‐Epn), parenchyma (Fb‐Par) and skin (Fb‐Skn)] were cultured on 24‐well plates coated with poly‐l‐lysine (Sigma‐Aldrich) in the Fb culture medium. The seeding cell densities of Fb‐Epn, Fb‐Par and Fb‐Par were adjusted to 0.8 × 10^4^, 2.0 × 10^4^ and 1.6 × 10^4^/cm^2^, respectively, such that a comparable 100% confluency was achieved when starting the DRG neuron culture the next day. On the next day, the Fb culture medium was removed, and DRG neurons were seeded onto them at a cell density of 5.0 × 10^3^ cells/cm^2^ with the neuron culture medium described above, followed by fixation with 4% PFA 48 h later. Three wells per condition were obtained.

In the second method, the Fb were separated on the insert, allowing only factors that were soluble from Fb to be available to the neurons below. Each type of Fb was cultured on a poly‐carbonate insert (0.4 μm pore size; catalogue no. 140620; Thermo Fisher Scientific) coated with poly‐l‐lysine at the same density as in the first method. On the next day, DRG neurons were seeded on the 24‐well plate coated with poly‐l‐lysine with the neuron culture medium, followed by fixation with 4% PFA 48 h later. Six wells per condition were obtained.

### 2.6 Culture of human endothelial cells

An immortalized human brain capillary cell line, hCMEC/D3 endothelial cells, were purchased from Sigma‐Aldrich. Cells from passages 32–35 were grown as described previously (Suzuki, Nakagawa, et al., 2022; Weksler et al., 2005). Cells were plated at a density of 5000 cells/cm^2^ in 24‐well plates, coated with 0.1 mg/ml collagen type 1 (Sigma‐Aldrich) in 400 μl endothelial growth basal medium‐2 (EBM‐2; Lonza, Switzerland) with 2.5% FBS. After 24 h, cells were fixed with 4% PFA for immunolabelling against Cd31.

### 2.7 Immunolabelling of cultured cells

**FIGURE 1:**
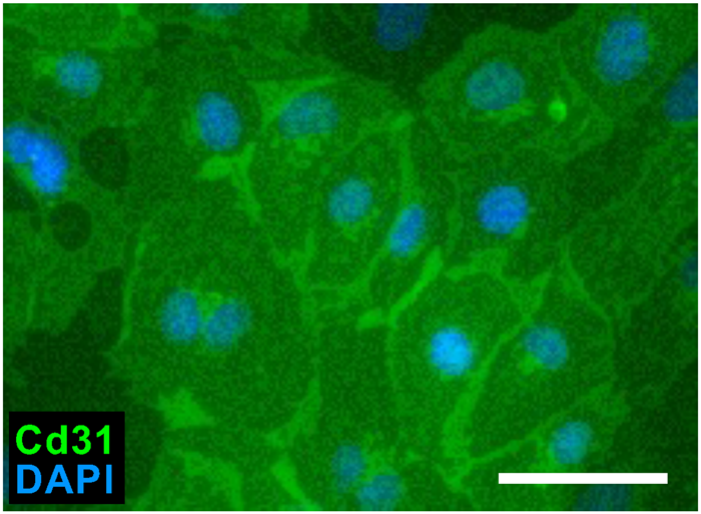
Cd31 immunolabelling of endothelial cells. Cells of the human brain endothelial cell line hCMEC/D3 were immunolabelled with antibody against Cd31 (goat, 1:100, catalogue no. AF3628; R&D Systems) and 4′,6‐diamidino‐2‐phenylindole (DAPI). Cd31 immunoreactivity was detected in all endothelial cells. Scale bar: 100 μm.

Fixed cells were blocked in Tris‐buffered saline (TBS) containing 5% horse serum (Thermo Fisher Scientific) and 0.125% Triton X (Sigma‐Aldrich), followed by an overnight incubation with primary antibodies at 4°C. After the cells had been washed with TBS three times, they were incubated for 1 h in secondary antibodies and 4′,6‐diamidino‐2‐phenylindole (DAPI; 100 ng/ml; Sigma‐Aldrich) at room temperature. The primary antibodies were anti‐type 1 collagen (goat, 1:200, catalogue no. 1310‐01; Southern Biotech), anti‐Thy1 Cd90 (mouse, 1:1000, catalogue no. ab225; Abcam, Cambridge, UK), anti‐Cd31 (goat, 1:100, catalogue no. AF3628; R&D systems), anti‐S100β (rabbit, 1:200, catalogue no. Ab52642; Abcam) and anti‐β3‐tubulin (mouse, 1:1000, catalogue no. 801202; BioLegend, San Diego, CA, USA). The secondary antibodies were Alexa 488‐ or 647‐conjugated donkey antibodies (1:1000, catalogue no. 715‐545‐151, 711‐545‐152 or 705‐605‐147; Jackson Immunoresearch, West Grove, PA, USA). Previous studies confirmed the specificity of immunolabelling against S100β (Endo et al., 2022; Endo, Kadoya, Kawamura, et al., 2019; Endo, Kadoya, Suzuki, et al., 2019; Ma et al., 2020; Meyer zu Reckendorf et al., 2020; Suzuki, Kadoya, et al., 2022), and that against Cd31 was verified by the immunolabelling of hCMEC/D3 cells (Figure 1) and previous studies (Zhang et al., 2020, 2021).

### 2.8 Neurite outgrowth assay

Immunolabelled images were obtained with an all‐in‐one fluorescence microscope (BZ‐ X710; Keyence, Osaka, Japan) using a ×20 objective lens. Neurites were detected by β3‐tubulin labelling, traced and measured with ImageJ software (Schneider et al., 2012) with the plugin software NeuronJ, as described previously (Kadoya et al., 2009; Meijering et al., 2004). To avoid the effect of interactions between neurons, if neurites touched the neurites of other neurons, these neurons were excluded from the analysis. Neurites that were ≥50 μm in length were defined as elongating neurites (Omura et al., 2015; Painter et al., 2014). At least 50 neurons were randomly selected per well, and the averages of the longest neurites and the percentage of neurons with elongating neurites were calculated.

### 2.9 Animal surgery and immunolabelling

Six 10‐ to 20‐week‐old rats (three males and three females) were anaesthetized by i.p. injection of a mixture of ketamine (75–100 mg/kg) and medetomidine (0.5 mg/kg). Animals were placed in the prone position, and a 4 cm longitudinal skin incision was made on the left posterior thigh. The sciatic nerve was fully exposed and was transected with scissors (*n* = 3, a male and two females) or crushed with micro‐mosquito forceps (*n* = 3, two males and a female) at the mid‐thigh. The operated side was dysfunctional, but it was unilateral, and there were no problems with food intake. For postoperative analgesia, buprenorphine (0.05 mg/kg) was administered s.c. four times during 3 days postoperatively. Seven days after the injury, the rats were anaesthetized again with a mixture of ketamine and medetomidine, followed by perfusion with 4% PFA in 0.1 M phosphate‐buffered saline. Dissected nerves were placed in 4% PFA overnight, then in 30% sucrose solution for ≥3 days.

Nerves were sectioned longitudinally at 10 μm thickness using a cryostat (Leica, Wetzlar, Germany). One out of nine sections was used for immunolabelling. The sections were incubated overnight with primary antibodies against pan‐neurofilament (mouse, 1:1000, catalogue no. 837904; BioLegend) and type 1 collagen (goat, 1:200, catalogue no. 1310‐01: SouthernBiotech) at 4°C. After washing with TBS, the sections were then incubated in Alexa‐488‐conjugated donkey anti‐mouse (1:1000, catalogue no. 715‐545‐151; Jackson Immunoresearch), Alexa‐647‐conjugated donkey anti‐goat (1:1000, catalogue no. 705‐605‐147; Jackson Immunoresearch) and DAPI for 1 h at room temperature.

### 2.10 RNA sequence analysis

RNA was extracted from a total of nine samples (three samples for each type of Fb) using an RNeasy plus mini kit (Qiagen, The Netherlands) according to the manufacturer's instructions. Total RNA integrity and quality were evaluated on an Aglient 2100 bioanalyzer (Aglient Technologies, Santa Clara, CA, USA), and samples of ≥1 μg with an RNA integrity number (RIN) of at least seven were used for the RNA‐seq. Each library was prepared using the NEBNext Poly(A) mRNA Magnetic Isolation Module (catalogue no. E7490), an NEBNext UltraTMII Directional RNA Library Prep Kit (catalogue no. E7760), and paired‐end reads (150 bp) were obtained on an Illumina NovaSeq 6000 (Illumina). Reads were aligned to the *Rattus norvegicus* genome rn6, and their quality was checked with FastQC (https://www.bioinformatics.babraham.ac.uk/projects/fastqc/). The average quality scores obtained with FastQC according to the position of bases in each read were all below an error rate of 0.001, indicating high quality that was classified as entirely normal. Sequence reads were trimmed with Trimmomatic (http://www.usadellab.org/cms/?page=trimmomatic) to remove low‐quality read terminals, adapter sequences and short reads. Specifically, we removed reads with a quality score of <20 at the beginning or the end of the read, or reads with a quality score of <15 on average by sliding the quality score by 4 bases (Bolger et al., 2014). In addition, reads that were <36 bp as a result of such trimming were removed. After these operations, sequence reads were mapped to the reference genome using HISAT2 (http://daehwankimlab.github.io/hisat2/), and all samples showed a high mapping rate of >97%. Obtained reads were normalized as transcripts per kilobase million (TPM) (Wagner et al., 2012; Zhao et al., 2020). Genes with a *P*‐value < 0.01 and a log_2_ fold change (FC) > 2 were defined as differentially expressed genes (DE genes). GeneOntology (GO) and KEGG (Kyoto Encyclopedia of Genes and Genome) pathway analyses were performed using the DAVID (https://david.ncifcrf.gov/) database. Heat maps were created using the R Stats package (https://rdocumentation.org/packages/stats/versions/3.6.1).

### 2.11 Statistics

The normality of the data distribution was assessed by the Shapiro–Wilk test, and all data were found to be normally distributed. Multiple‐group comparisons were performed with a one‐way ANOVA with the Tukey–Kramer test. All statistics were performed with JMP software (SAS, Cary, NC, USA), with a designated significance level of 95%. Data are presented as the mean value ± SD.

## 3 RESULTS

### 3.1 Nerve‐derived Fb have a greater neurite‐promoting effect than skin‐derived Fb

**FIGURE 2:**
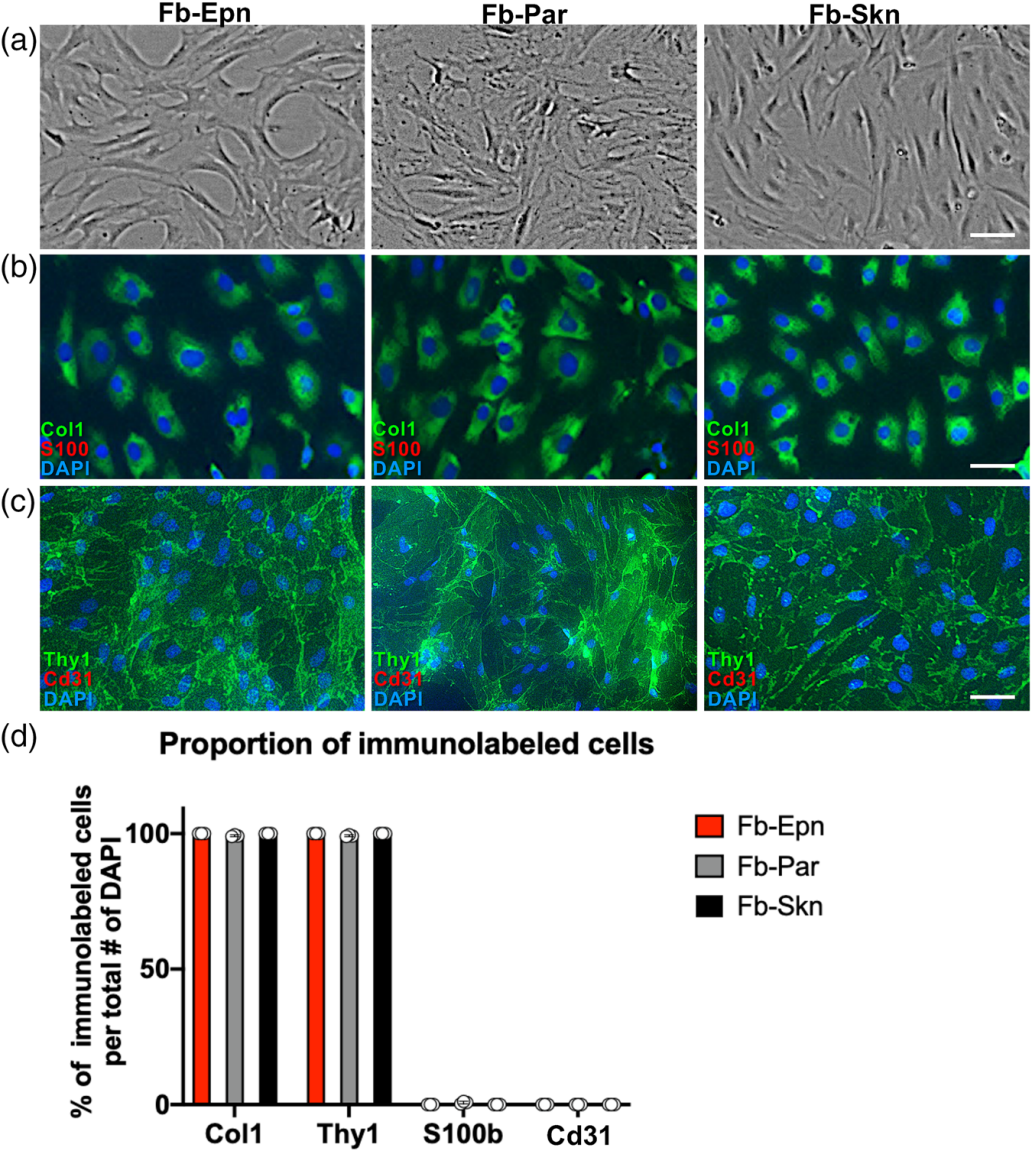
Prepared fibroblasts (Fb) demonstrated high purity. (a) Representative phase‐contrast images of epineurium‐derived fibroblasts (Fb‐Epn), parenchyma‐derived fibroblasts (Fb‐Par) and skin‐derived fibroblasts (Fb‐Skn). Scale bar: 50 μm. (b) Representative images of Fb immunolabelled against type 1 collagen (Col1), S100b and 4′,6‐diamidino‐2‐phenylindole (DAPI). Scale bar: 50 μm. (c) Representative images of Fb immunolabelled against Thy1, Cd31 and DAPI. Scale bar: 50 μm. (d) Quantification of the number of immunolabelled cells as a percentage of the total number of DAPI‐labelled cells. An individual dot indicates one well. All cells were labelled with Col1 and Thy1, and immunoreactivity for S100b and Cd31 was negligible. *n* = 3 wells per group.

All three types of Fb demonstrated a similar morphology (Figure 2a) and expressed Fb markers such as type 1 collagen and Thy1 (Figure 2b–d), but the immunoreactivity of SCs and vascular ECs was negligible (Figure 2b–d), indicating the high purity of the cultured Fb. Although SCs and ECs are present in peripheral nerves and are adherent cells, the primary Fb that were cultured from rat sciatic nerves had a high purity. This can be attributed to the fact that the cell culture medium was discarded 1 h after seeding cells and also indicates that Fb have a significantly higher adhesive property than SCs and vascular ECs.

**FIGURE 3:**
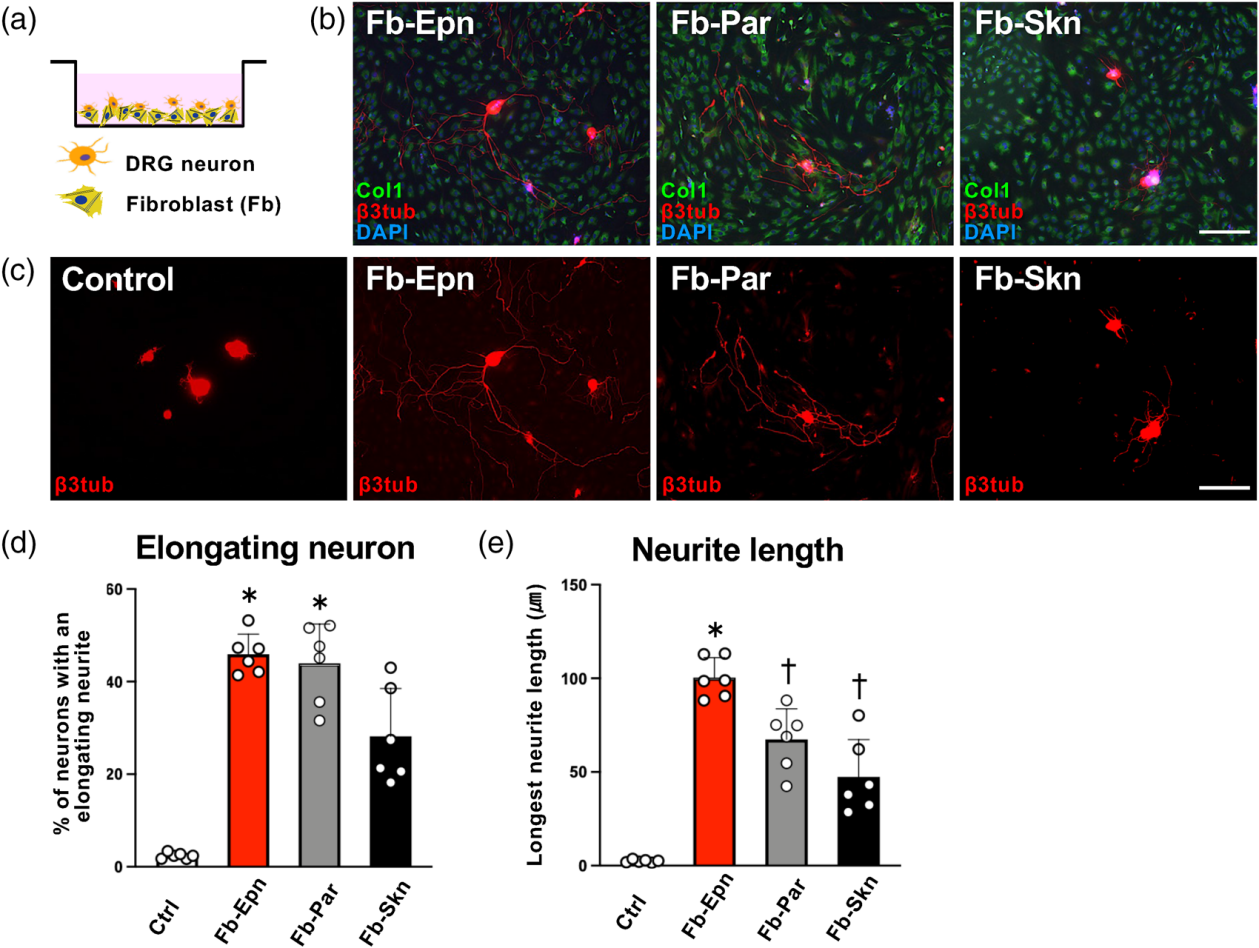
Nerve‐derived fibroblasts (Fb) promoted neurite outgrowth of adult dorsal root ganglion (DRG) neurons. (a) The schematic diagram depicts DRG neurons seeded on Fb. (b) Representative images of DRG neurons and Fb triple‐labelled against type 1 collagen (Col1), β3‐tubulin (β3tub) and 4′,6‐diamidino‐2‐phenylindole (DAPI). Scale bar: 150 μm. (c) Representative images of DRG neurons labelled against β3tub. Neurons co‐cultured with Fb have longer neurites than the non‐co‐cultured neurons. Scale bar: 150 μm. (d) Quantification of the percentage of DRG neurons with an elongating neurite. An individual dot indicates one well. Epineurium‐derived fibroblasts (Fb‐Epn) and parenchyma‐derived fibroblasts (Fb‐Par) induced significantly more elongating neurons than control. *n* = 6 wells per condition. One‐way ANOVA (*F* = 3.09, *P* < 0.0001) with the Tukey–Kramer test. ^*^ *P* < 0.05 compared with control. (e) Quantification of the longest neurite of DRG neurons. Individual dots indicate one well. The Fb‐Epn induced the longest neurite among all groups, and Fb‐Par and skin‐derived fibroblasts (Fb‐Skn) induced longer neurites than control. *n* = 6 wells per condition. One‐way ANOVA (*F* = 3.09, *P* < 0.0001) with the Tukey–Kramer test. ^*^ *P* < 0.05 compared with others; ^†^ *P* < 0.05 compared with the control.

One day after the Fb seeding, adult DRG neurons were cultured on them (Figure 3a), followed by fixation 2 days later. A few neurons on the poly‐l‐lysine‐coated plate had elongated neurites, but significantly more DRG neurons on Fb induced elongated neurites [Figure 3b–d; one‐way ANOVA (*F* = 3.09, *P* < 0.0001) with the Tukey–Kramer test, *P* < 0.05]. Among the three types of Fb, only 28.2% of the neurons had elongating neurites in the Fb‐Skn co‐cultures, whereas 45.9 and 43.9% of the neurons had elongating neurites in the Fb‐Epn and Fb‐Par co‐cultures (Figure 3d). Quantitative measurements of the average length of the longest neurite indicated that the neurons co‐cultured with Fb‐Epn were the longest (100.4 μm), followed by neurons that were co‐cultured with Fb‐Par (67.4 μm) and Fb‐Skn (47.4 μm) (Figure 3e). These results indicate that Fb stimulate the outgrowth of neurites from DRG neurons, that PNS‐derived Fb have a substantially greater neurite‐promoting effect than Fb‐Skn, and that Fb‐Epn stimulate neurite outgrowth more effectively than Fb‐Par.

### 3.2 Fibroblasts promote neurite outgrowth in adult DRG neurons by both contact‐mediated and soluble factors

**FIGURE 4:**
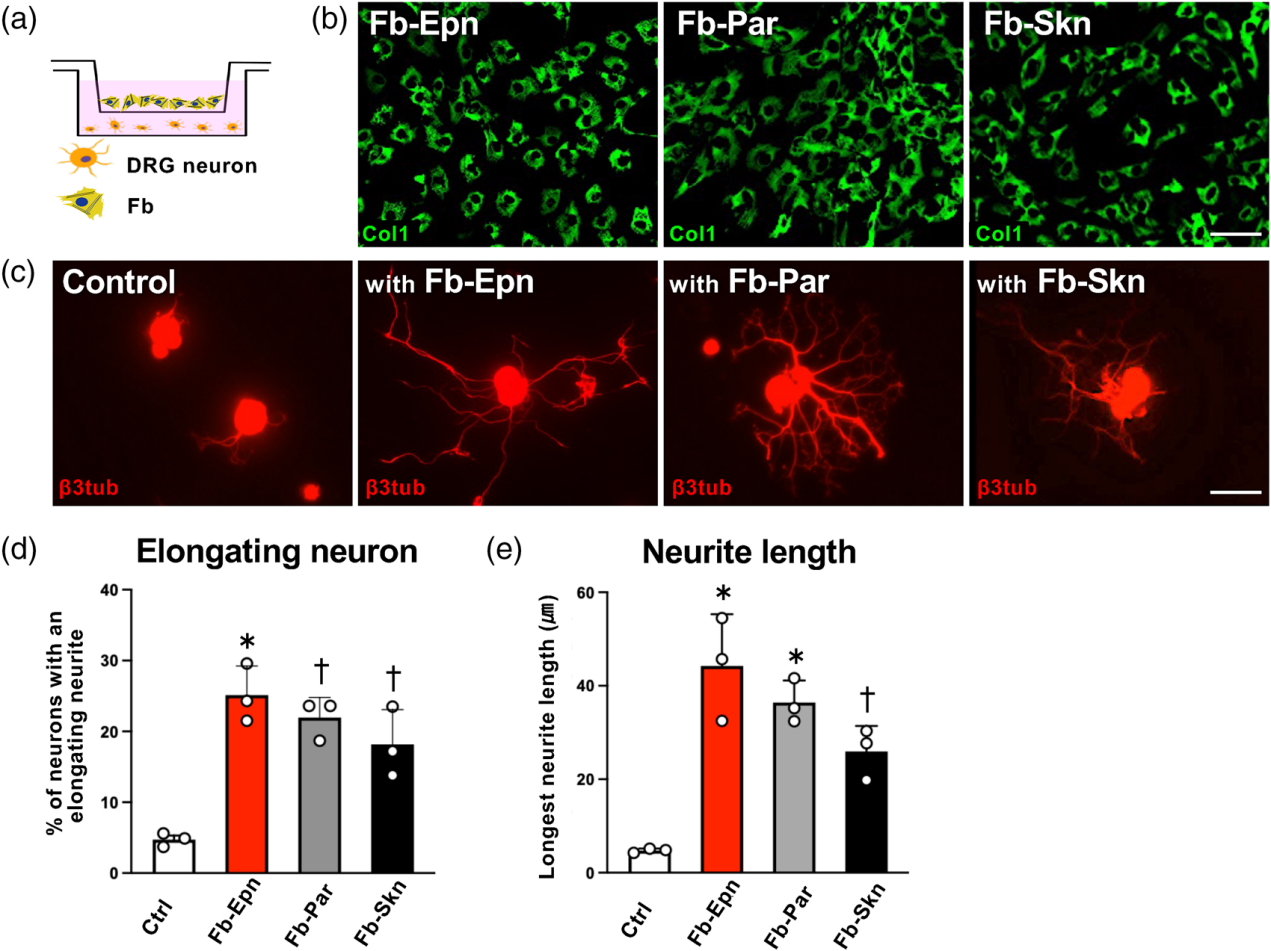
Factors secreted from the nerve‐derived fibroblasts (Fb) promoted neurite outgrowth of adult dorsal root ganglion (DRG) neurons. (a) The schematic diagram depicts DRG neurons separated from Fb in the same well. (b) Representative images of Fb cultured on an insert. The Fb were immunolabelled with type 1 collagen (Col1). Scale bar: 100 μm. (c) Representative images of DRG neurons labelled against β3‐tubulin (β3tub). Neurons co‐cultured with Fb have longer neurites than the non‐co‐cultured neurons. Scale bar: 50 μm. (d) Quantification of the percentage of DRG neurons with an elongating neurite. An individual dot indicates one well. Epineurium‐derived fibroblasts (Fb‐Epn) induced significantly more elongating neurons than any other conditions, and parenchyma‐derived fibroblasts (Fb‐Par) and skin‐derived fibroblasts (Fb‐Skn) induced more than control. *n* = 3 wells per condition. One‐way ANOVA (*F* = 4.06, *P* = 0.0002) with the Tukey–Kramer test. ^*^ *P* < 0.05 compared with others. (e) Quantification of the longest neurite of DRG neurons. An individual dot indicates one well. The Fb‐Epn and Fb‐Par induced significantly longer neurites than Fb‐Skn and control, and Fb‐Skn induced longer neurites than control. *n* = 3 wells per condition. One‐way ANOVA (*F* = 4.06, *P* = 0.0003) with the Tukey–Kramer test. ^*^ *P* < 0.05 compared with Fb‐Skn and control; ^†^ *P* < 0.05 compared with control.

Given that neurons were seeded directly onto Fb in the preceding experiments, the issue of whether the enhanced neurite outgrowth was attributable to either soluble factors or cell‐surface factors from Fb was unclear. To clarify this, we separated co‐cultured Fb from neurons by placing the Fb onto inserts in the same well (Figure 4a,b). After 2 days, all types of Fb significantly increased the percentage of neurons with elongating neurites compared with the control [one‐way ANOVA (*F* = 4.06, *P* = 0.0002) with the Tukey–Kramer test, *P* < 0.05], which had no co‐cultured Fb (Figure 4c). The Fb‐Epn had the highest percentage of neurons with elongating neurites, followed by Fb‐Par and Fb‐Skn; 4.7% for the control, and 24.4, 22.3 and 18.4% for the Fb‐Epn, Fb‐Par and Fb‐Skn, respectively (Figure 4d). In addition, quantification of the average length of the longest neurite showed that the neurons that had been co‐cultured with Fb‐Epn were the longest, followed by neurons co‐cultured with Fb‐Par and Fb‐Skn (Figure 4e); 4.7 μm for the control, and 44.3, 36.5 and 25.9 μm for the Fb‐Epn, Fb‐Par and Fb‐Skn, respectively.

When these numbers were compared with those of the preceding experiment, there was a 53, 51 and 64% reduction in Fb‐Epn, Fb‐Par and Fb‐Skn, respectively, in the neurons with elongating neurites and a 45.3, 56.0 and 45.8% reduction in Fb‐Epn, Fb‐Par and Fb‐Skn, respectively, in the longest neurite length. These findings indicate that the neurite‐promoting effect of Fb‐Epn and Fb‐Par can be attributed to soluble and cell‐surface factors to the same degree.

### 3.3 Fibroblasts show no close association with regenerating axons after PNI

**FIGURE 5:**
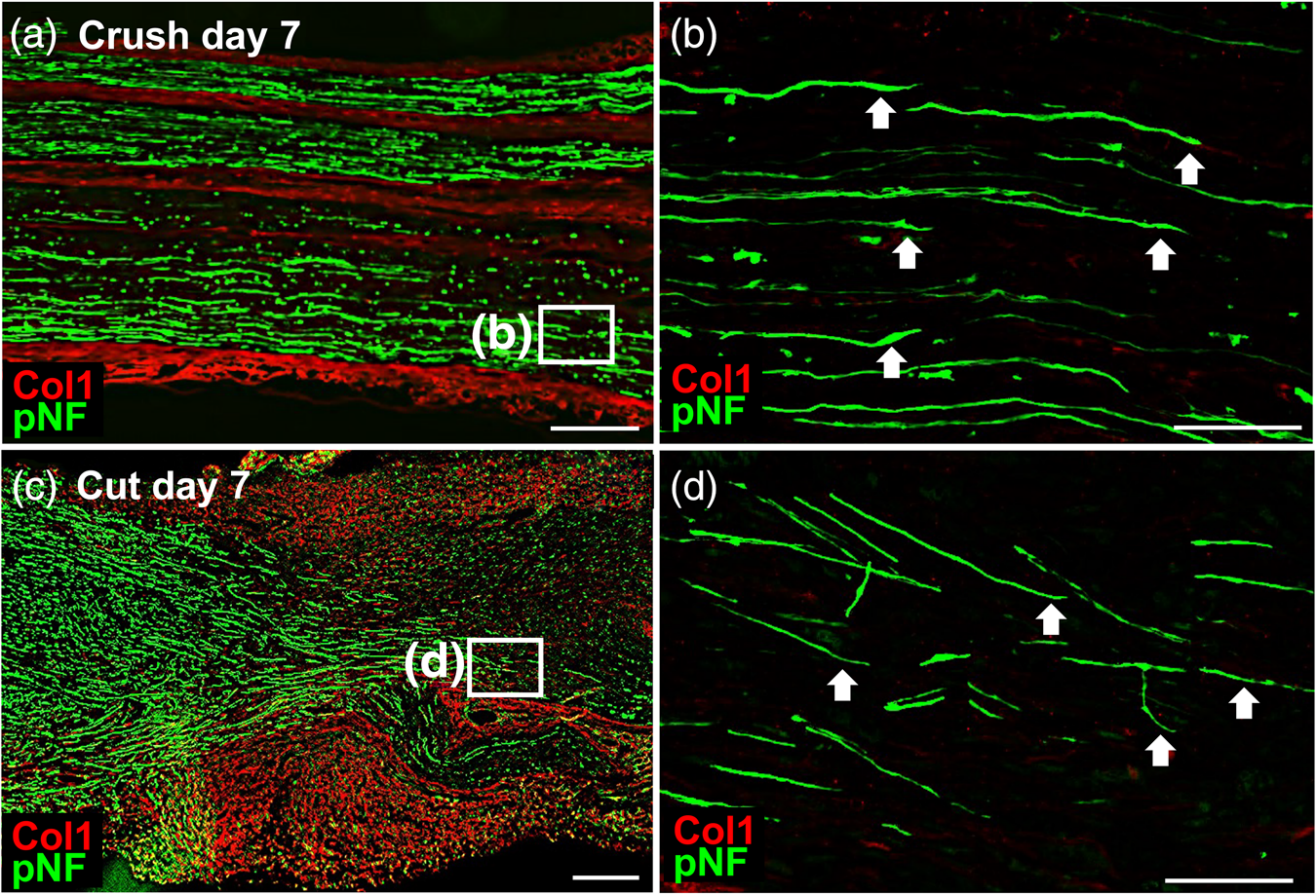
Fibroblasts (Fb) are not closely associated with regenerating axons after peripheral nerve injury. (a) Representative low‐magnification image of a longitudinal section of rat sciatic nerve at 7 days after a crush injury, 1.8 mm distal to the injury site. Left is proximal. Double immunolabelling with type 1 collagen (Col1) and pan‐neurofilament (pNF) shows Fb and regenerating axons. Scale bar: 300 μm. (b) The magnified image of the boxed area in (a). Arrows indicate tips of regenerating axons. No immunoreactivity for Col1 was observed, indicating the absence of a close association of Fb with regenerating axons. Scale bar: 50 μm. (c) Representative low‐magnification image of injury site in a longitudinal section of rat sciatic nerve at 7 days after a transection injury. Left is proximal. Double immunolabelling with Col1 and pNF shows many accumulating Fb and regenerating axons. Scale bar: 300 μm. (d) The magnified image of the boxed area in (c). Arrows indicate tips of regenerating axons. No immunoreactivity for Col1 was observed, indicating the absence of a close association of Fb with regenerating axons. Scale bar: 50 μm.

Given that it was shown that Fb have the ability to promote axon regeneration, we aimed to clarify the possibility that Fb are directly involved in axon regeneration. We investigated the relationship between the positions of Fb and the regenerating axons using two injury models, namely, a crush injury and a transection injury. At 7 days after the crush injury, regenerating axons were observed in the area of Wallerian degeneration, and Fb were located mainly at the epineurium and not at all around the regenerating axons (Figure 5a,b). At 7 days after the transection injury, numerous Fb were observed at the injury site (Figure 5c), and regenerating axons were observed to be traversing the injury site (Figure 5c,d), but none of the Fb was closely associated with regenerating axons (Figure 5d). These findings suggest that, although cell‐surface factors that are expressed by Fb have the capability to promote axon regeneration, only soluble factors derived from them are available to regenerating axons after PNI.

### 3.4 Molecular profiles of the three types of Fb are distinctly different

**FIGURE 6:**
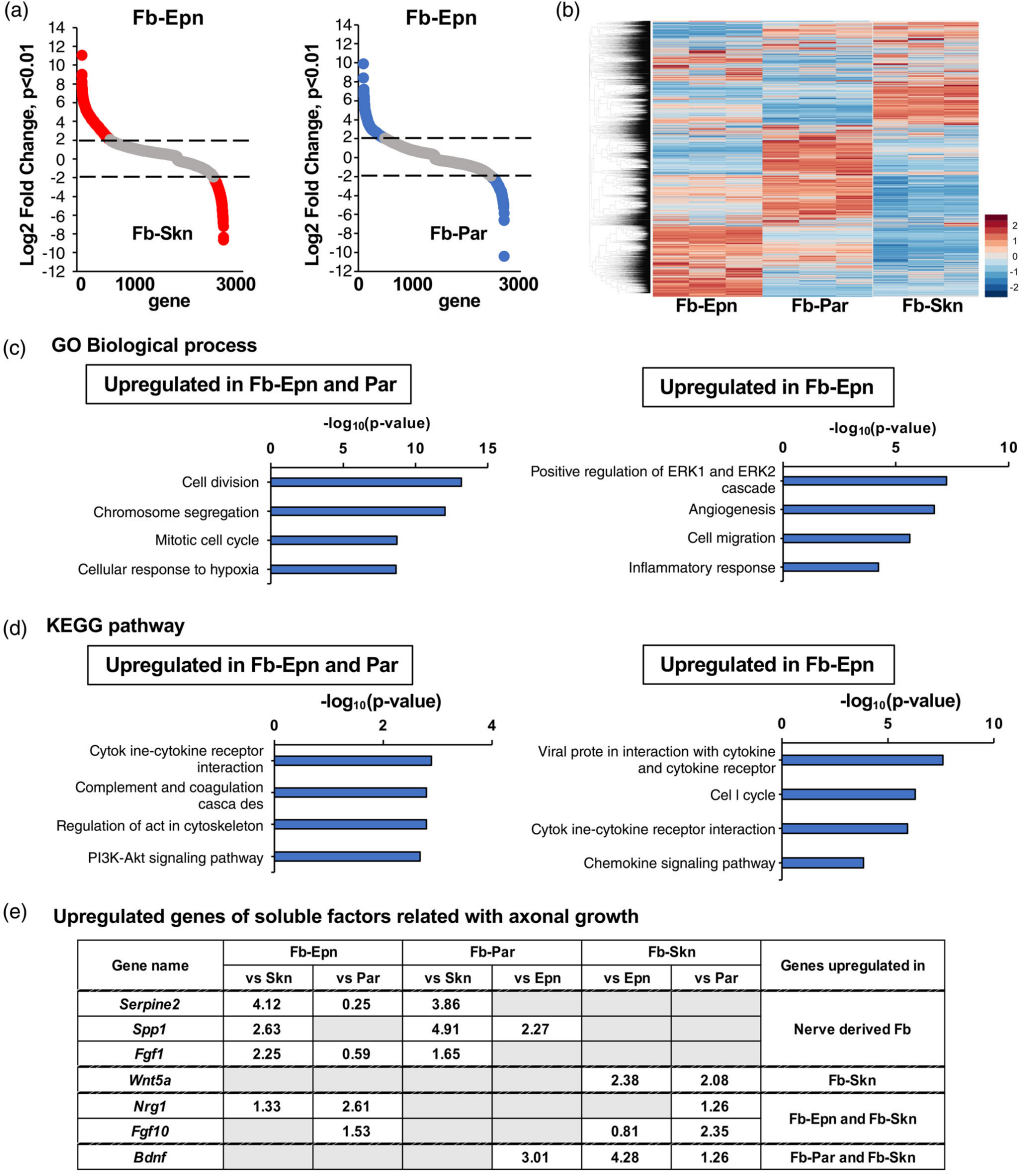
Molecular profiles of three types of fibroblasts (Fb) are distinctly different. (a) Scatterplot analysis of genes with significantly upregulated or downregulated expression in epineurium‐derived fibroblasts (Fb‐Epn) and parenchyma‐derived fibroblasts (Fb‐Par), and in Fb‐Epn and skin‐derived fibroblasts (Fb‐Skn). Red and blue circles indicate genes with log_2_ fold change >2 and <−2. Transcriptional expression levels of Fb‐Epn were significantly different from those of Fb‐Skn and Fb‐Par, at 2811 and 2681 genes, respectively (*P* < 0.01), and changed >4‐fold from those of Fb‐Skn and Fb‐Par, at 753 and 652 genes, respectively (*P* < 0.01). *n* = 3 samples per group. (b) Heatmap of gene expressions in Fb‐Epn, Fb‐Par and Fb‐Skn. Red and blue indicate the highest and lowest respective relative levels of gene expressions. (c) Top four enriched gene ontology (GO) terms of biological processes. The left panel shows GO analysis performed for 196 genes significantly upregulated in Fb‐Epn and Par‐Fb relative to Fb‐Skn. The right panel shows 403 genes that are significantly upregulated in Fb‐Epn relative to Fb‐Par. The horizontal axis shows −log_10_ (*P*‐value). (d) Top four KEGG pathway analysis terms. The same gene sets were used as in (c). The left panel shows the analysis of genes significantly upregulated in Fb‐Epn and Fb‐Par relative to Fb‐Skn. The right panel shows the analysis of genes significantly upregulated in Fb‐Epn relative to Fb‐Par. The horizontal axis shows −log_10_ (*P*‐value). (e) Seven genes were identified as differentially expressed (DE) genes upregulated in specific Fb among 29 genes of soluble factors known to be related to axonal growth (Table 1). Genes with a *P*‐value < 0.01 and a log_2_ fold change >2 were defined as DE genes. The log_2_ ratio of gene expression to another Fb is listed.

To explore the molecular mechanism underlying the neurite outgrowth‐promoting effects of Fb, we performed RNA sequence analysis on Fb. A comparison of the gene expressions revealed that the transcriptional expression levels were significantly different in 2811 genes (*P* < 0.01) between Fb‐Epn and Fb‐Skn and that 753 genes were changed by >4‐fold (*P* < 0.01; Figure 6a). Between Fb‐Epn and Fb‐Par, the expression levels of 2681 genes were significantly different (*P* < 0.01), and 652 genes were changed by >4‐fold (*P* < 0.01; Figure 6b). Heat maps of the hierarchical clustering of gene expressions also showed an apparent difference in gene expression profiles between the groups, and a high degree of similarity between samples was observed within the same groups (Figure 6b). These findings clearly indicate that the molecular profiles of these three Fb are distinctly different.

GO analyses were performed on 196 genes that were significantly upregulated in both Fb‐Epn and Fb‐Par (nerve‐derived Fb) relative to Fb‐Skn (*P* < 0.01), and cell proliferation‐related terms, such as cell division, chromosome segregation and mitotic cell cycle, were identified in nerve tissue‐derived Fb (Figure 6c, left panel), suggesting that these cells have greater proliferative properties. KEGG analyses revealed an increase in cytokine–cytokine receptor interactions and complement and coagulation cascades in the nerve‐derived Fb (Figure 6d, left panel), suggesting that they function in the maintenance and regulation of homeostasis and the mobilization of inflammatory cells, both of which potentially have important roles in axon regeneration and the repair process after PNI (Bajic et al., 2015; Siqueira Mietto et al., 2015; Turner et al., 2014).

Another GO analysis was performed on 403 genes that were significantly upregulated in Fb‐Epn relative to Fb‐Par (*P* < 0.01), and angiogenesis, cell migration and inflammatory responses were identified (Figure 6c, right panel), suggesting that Fb‐Epn function for angiogenesis by migrating and supporting inflammation. A KEGG analysis identified cell cycle, cytokine–cytokine receptor interactions and chemokine signalling pathways in Fb‐Epn, suggesting that Fb‐Epn might be involved in an injury response by inducing the production of immune cells (Thelen & Stein, 2008) and regulating the inflammatory response.

Given that soluble factors from Fb promoted neurite outgrowth (Figure 4d,e) and could contribute to axon regeneration after PNI, gene expressions of 29 soluble factors known to be related with axonal growth were investigated (Table 1). Among them, seven genes were identified as DE genes upregulated in specific Fb (Figure 6e). *Serpine2* (Becerra et al., 1995; Guenther et al., 1985), *Spp1* (Wright et al., 2014) and *Fgf1* (Hossain & Morest, 2000; Lin et al., 2009) were upregulated in peripheral nerve‐derived Fb. Especially, *Serpine2* and *Fgf1* were expressed at higher levels in Fb‐Epn than in Fb‐Par. Fb‐Skn upregulated *Wnt5a* (Yang et al., 2011). *Nrg1* and *Fgf10* were upregulated in Fb‐Epn and Fb‐Skn. *Bdnf* (Chen et al., 2006; Davies, 2000) was upregulated the most in Fb‐Skn, followed by Fb‐Par. *Thbs1* (Bray et al., 2019), *Serpine1* (Parmar et al., 2002; Soeda et al., 2004) and *Lgals1* (Horie et al., 1999; McGraw et al., 2004) were expressed at high levels in all types of Fb, whereas expressions of *Egf*, *Fgf4*, *Fgf8*, *Fgf21*, *Nrg2*, *Nrg4* and *Ntf3* were not detected or found at very low levels (Table 1). These findings suggest that the neurite‐promoting effect of Fb is mediated by soluble factors that are unique to each Fb and that the superior capacity of Fb‐Epn in neurite outgrowth could be mediated by a synergetic effect of multiple soluble factors.

**TABLE 1:** List of genes of soluble factors known to be related to axonal growth.

Gene symbol or name	Average TPM			*q* value		
	Fb‐Epn	Fb‐Par	Fb‐Skn	Fb‐Epn vs. Fb‐Par	Fb‐Epn vs. Fb‐Skn	Fb‐Par vs. Fb‐Skn
*Bdnf*	0.73	5.89	14.15	18.34	47.66	29.31
*Cntf*	9.01	7.53	3.23	3.55	13.90	10.35
*Egf*	0.24	0.35	0.23	0.94	0.13	1.07
*Fgf1*	8.71	5.75	1.83	11.31	26.30	14.98
*Fgf10*	25.29	8.70	44.43	19.64	22.66	42.30
*Fgf2*	13.32	20.12	15.56	4.33	1.42	2.91
*Fgf21*	0.00	0.00	0.42	0.00	8.63	8.63
*Fgf4*	0.00	0.00	0.00	NA	NA	NA
*Fgf8*	0.00	0.00	0.00	NA	NA	NA
*Fgf9*	2.56	0.55	1.49	7.84	4.17	3.67
*Gdnf*	3.25	1.55	6.14	1.54	2.62	4.16
*Hgf*	1.46	0.15	1.05	9.88	3.13	6.75
*Igf1*	48.32	102.88	45.71	35.69	1.71	37.40
*Lgals1*	2184.48	2957.46	2814.71	6.23	5.08	1.15
*Lif*	67.62	27.56	18.87	42.53	51.76	9.23
*Ngf*	18.22	21.65	34.54	3.73	17.75	14.02
*Nrg1*	14.69	2.41	5.81	42.45	30.69	11.76
*Nrg2*	0.04	0.07	0.02	0.59	0.47	1.06
*Nrg4*	0.26	0.07	0.14	1.64	1.07	0.58
*Ntf3*	0.00	0.00	0.00	NA	NA	NA
*Ntf4*	2.37	1.78	1.38	2.27	3.81	1.54
Osteopontin	443.14	2149.31	71.15	77.57	16.91	94.48
Pleiotrophin	4.06	1.25	0.15	6.78	9.42	2.64
*Serpine1*	4083.61	2338.29	1893.87	54.59	68.49	13.90
*Serpine2*	94.10	78.60	5.41	6.71	38.41	31.70
*Tgfb1*	131.31	142.24	119.29	2.12	2.33	4.45
*Thbs1*	12522.50	6601.84	5960.27	35.44	39.28	3.84
*Vegfa*	314.44	101.42	307.38	74.06	2.45	71.60
*Wnt5a*	8.65	10.69	45.28	1.72	30.80	29.08

Abbreviations: Fb‐Epn, epineurium‐derived fibroblasts; NA, not assessed; Fb‐Par, parenchyma‐derived fibroblasts; Fb‐Skn, skin‐derived fibroblasts; TPM, transcripts per kilobase million.

*Note*: The average TPM of each Fb and *q* value are summarized. One‐way ANOVA (*F* = 5.14, *P* < 0.01) with the Tukey–Kramer test. A value of *q* > 10.62 means that there is a statistical difference (*P* < 0.01).

Finally, to explore a shared mechanism among Fb for axonal growth, genes of soluble factors were listed from the top 100 genes highly expressed in each Fb (Table 2). Out of 29 genes listed in total, 15 genes, including *Thbs1*, *Serpine1* and *Lgals1*, were shared among all types of Fb, suggesting that these factors might have the capability to stimulate neurite outgrowth of adult DRG neurons directly or cooperatively with other soluble factors.

**TABLE 2:** List of soluble factor genes ranked within the top 100 highly upregulated genes in each Fb type.

Fb‐Epn	Fb‐Par	Fb‐Skn
*Col1a1* *	*Col1a1* *	*Col1a1* *
*Col3a1* *	*Mgp*	*Ccn2* *
*Thbs1* *	*Col3a1* *	*Thbs1* *
*Ccn2* *	*Thbs1* *	*Col3a1* *
*Fn1* *	*Ccn2* *	*Bgn* *
*Bgn* *	*Fth1* *	*Col12a1*
*Serpine1* *	*Bgn* *	*Col5a2* *
*Ccdc80* *	*Fn1* *	*Lgals1* *
*Fth1* *	*Col4a1*	*Fstl1* *
*Col5a2* *	*Fstl1* *	*Fth1* *
*Il1rl1*	*Lgals1* *	*Fn1* *
*Fbln2*	*Dcn*	*Ccdc80* *
*Lgals1* *	*Col4a2*	*Timp2* *
*Ccn1*	*Ccdc80* *	*Thbs2*
*Mgp*	*Serpine1* *	*Ppia* *
*Timp1*	*Col6a1*	*Serpine1* *
*Fstl1* *	*Col5a2* *	*Fbln2*
*F3*	*Txn1*	*Pcolce*
*Ppia* *	*Spp1*	*Calu* *
*Postn*	*Postn*	*Ccn1*
*Timp2* *	*Timp2* *	*Mmp2*
*Calr*	*Wfdc1*	*Calr*
*Txn1*	*Ctsl*	*Col5a1*
*Mmp2*	*Ppia* *	
*Calu* *	*Col8a1*	
*Tnc*	*Col6a2*	
	*Pcolce*	
	*Lgfbp7*	
	*Calu* *	
	*Prss23*	
	*Fbn1*	

Abbreviations: Fb, fibroblasts; Fb‐Epn, epineurium‐derived fibroblasts; Fb‐Par, parenchyma‐derived fibroblasts; Fb‐Skn, skin‐derived fibroblasts; TPM, transcripts per kilobase million.

*Note*: Gene names are listed in order of decreasing TPM.

* A gene listed for all types of Fb.

## 4 DISCUSSION

The findings of the present study demonstrate that nerve‐derived Fb have a greater neurite‐promoting effect than that of Fb‐Skn and that Fb‐Epn stimulate neurite outgrowth more effectively than Fb‐Par. Although soluble and cell‐surface factors contributed to their neurite‐promoting effect evenly, in real circumstances after PNI, only the soluble factors from Fb are available to regenerating axons. Lastly, the molecular profiles of the three Fb were distinctly different, and the gene expression profiles of the soluble factors to promote neurite growth were unique to each Fb. These findings reveal that nerve‐derived Fb are functionally and molecularly different from skin‐derived Fb, and even in the nerve, Fb‐Epn differ from Fb‐Par in terms of the capacity for stimulating neurite outgrowth and the molecular profiles.

In the present study, nerve‐derived Fb were found to have a greater effect in terms of promoting the outgrowth of neurites in DRG neurons compared with Fb‐Skn. This superior effect can be attributed to a several fundamental anatomical facts. First, the axonal density in peripheral nerve is much higher than that in the skin. As a result, Fb functioning for axons could be present at higher levels in peripheral nerve than Fb in skin. Secondly, axons do not need to regenerate over a long distance after a skin injury, because skin is a target organ that is innervated by axons. On the contrary, axons in peripheral nerves need to regenerate over a long distance after PNI in order to reach a target organ. Therefore, axons in the skin might not need much support from neighbouring non‐neuronal cells, unlike axons in peripheral nerves. It should also be noted that, in the present study, we used an assay used for adult DRG neurons that contain numerous types of sensory neurons, because our goal was to understand the effects of Fb on PNI. If we could assess DRG neurons that are innervated exclusively in skin, it is possible that Fb‐Skn might have been found to show a greater neurite outgrowth effect than the results for the present experiment.

Generally, skin and nerves require different forms of repair after injury. When a defect is generated at the site of skin injury, Fb‐Skn secrete growth factors, such as nerve growth factor, vascular endothelial growth factor and platelet‐derived growth factor, to increase cell proliferation, stimulate the migration of vascular ECs and promote extracellular matrix production in order to fill the defect (Guerrero‐Juarez et al., 2019). In the case of PNI, a defect is also generated at the injury site, but it is subsequently filled mainly by SCs, with the support of ECs and Fb (Min et al., 2021). Axons then regenerate through the injury site by following bundles of SCs (Jessen et al., 2015) and continue to grow in the region of Wallerian degeneration, which is long, but has no tissue defect. Accordingly, the functions of Fb in the nerve after PNI appear to be different from those of Fb in the skin after an injury. Indeed, not only their neurite outgrowth‐promoting function, but also their molecular profiles were distinctly different from each other. The biological functions identified were also different. These findings support the conclusion that Fb at different anatomical locations have different functions, even in the PNS.

Given that the epineurium separates nervous parenchyma from surrounding tissues, it is reasonable to assume that the epineurium might function to prevent axons from growing ectopically and that Fb‐Epn might fail to stimulate neurite outgrowth. However, Fb‐Epn demonstrated a superior neurite‐promoting effect compared with that for Fb‐Par. This raises the possibility that Fb‐Epn could be more involved in axon regeneration than previously thought. Importantly, Fb were cultured in a damage‐signalling environment, because the Fb were prepared by enzymatic digestion and mechanical dissociation, indicating that the present investigation reflects more on injured conditions rather than a steady state. In vivo, the transection site is surrounded by abundant Fb, which could be derived from both Fb‐Epn and Fb‐Par (Figure 5c), supporting a scenario in which Fb‐Epn in an injured environment augment axonal growth at the site of the lesion.

Regarding the mechanism of axonal growth supported by Fb, soluble factors clearly showed an axon‐promoting effect. Nerve‐derived Fb upregulated three genes of soluble factors known to be related to axonal growth, namely glia‐derived nexin (GDN) (Becerra et al., 1995; Guenther et al., 1985), osteopontin (Wright et al., 2014) and Fgf1 (Hossain & Morest, 2000; Lin et al., 2009), whereas Fb‐Skn upregulated *Wnt5a* (Yang et al., 2011). Especially, Fb‐Epn upregulated genes of GDN, Fgf1 and neuregulin1 (Nrg1) significantly more than Fb‐Par and Fb‐Skn, suggesting that the superior capability of Fb‐Epn for neurite outgrowth might be mediated by the secretion of these factors. In PNS, GDN is secreted by SCs, DRG neurons and spinal motor neurons (Mansuy et al., 1993; Niclou et al., 1998; Schira et al., 2019). After PNI, increased expression of *Serpine2* was observed in SCs (Gill et al., 1998) and endoneurial mesenchymal cells (Kalinski et al., 2020), but the expression of *Serpine2* in epineurium is not known. Although GDN promotes neurite outgrowth of sympathetic neurons (Zurn et al., 1988) and neonatal DRG neurons (Hawkins & Seeds, 1986) and contributes to the repair processes after PNI by supporting proliferation and survival of SCs (Lino et al., 2007), its direct effect on regenerating axons remains to be determined. Fgf1 is present in almost all tissues and promotes reparative processes after injury in many organs (Dhlamini et al., 2022; Ornitz & Itoh, 2015). Furthermore, it stimulates neurite outgrowth of PC12 cells (Lin et al., 2009) and promotes axon regeneration after both spinal cord injury and PNI (Hsu et al., 2013; Li et al., 2018). Accordingly, Fgf1 appears to contribute to the effect of Fb‐Epn on neurite outgrowth of adult DRG neurons. Transmembrane Nrg1 is known to be expressed by axons during development to stimulate SCs to myelinate them (Newbern & Birchmeier, 2010). Nrg1 can stimulate neurite outgrowth of postnatal spinal motor neurons and DRG sensory neurons (Mòdol‐Caballero et al., 2018) . Although both axons and SCs express different isoforms of Nrg1 to support remyelination and axonal growth after PNI (Gambarotta et al., 2013; Stassart et al., 2013), and soluble Nrg1 is highly expressed by nerve‐derived Fb (Fornasari et al., 2020), its direct effect on regenerating axons needs to be explored further. Given that Fb‐Epn did not attract much attention as key players in the repair process after PNI, the detailed information about them is largely lacking. Future studies need to clarify the specific role of Fb‐Epn in axon regeneration and the repair process after PNI, and the molecular features obtained in the present study could help with this.

Concerning the shared mechanism among tested Fb, 15 genes of soluble factors were found to be upregulated in all types of Fb, including genes of thrombospondin 1 (Tsp1), neuroserpin and galectin1. Tsp1 is a substrate for neurite adhesion and promotes axon regeneration after optic nerve injury (Neugebauer et al., 1991; O'Shea et al., 1991). Neuroserpin promotes neurite outgrowth of PC12 neurons (Parmar et al., 2002; Soeda et al., 2004), and galectin1 promotes axon regeneration after PNI (Horie & Kadoya, 2000). Therefore, these factors appear to underlie the axon‐promoting effect of all types of Fb, although gene upregulation does not necessarily mean enhanced protein secretion. The other 12 genes include typical Fb genes, such as *Col1a1*, *Col3a1* and *Fn1*, in addition to *Bgn*, which codes for biglycan, a small, leucine‐rich chondroitin sulfate proteoglycan (Diehl et al., 2021). Given that this matrix protein has never been explored as a substrate for axonal growth, it might be worth investigation.

The Fb‐Par can be categorized into perineurial Fb and endoneurial Fb by their anatomical locations, molecular features and developmental origins (Chen et al., 2021; Joseph et al., 2004). Therefore, it would be desirable to analyse them separately rather than as mixed populations of Fb‐Par. However, the perineurium attaches to the parenchyma tightly as a sheath, making its enzymatic and manual dissection technically difficult. In addition, given that cell surface markers of perineurial Fb and endoneurial Fb remain to be identified, a method of antibody‐mediated selection has not yet been developed. Interestingly, recent single‐cell transcriptome analysis proposed the possibility that endoneurial Fb were more important for promoting peripheral nerve regeneration than Fb‐Epn or perineurial Fb, based on the fact that endoneurial Fb had much higher number of significant DE genes than other Fb (Chen et al., 2021). Once an isolation method or cell‐specific manipulation method has been established, a future study is needed to elucidate the specific functions of perineurial Fb and endoneurial Fb after PNI.

Several studies have already investigated the molecular and functional characteristics of nerve‐derived Fb. One study found that nerve‐derived Fb expressed brain derived neurotrophic factor (Bdnf) in the proximal and distal stumps of the injured sciatic nerve (He et al., 2022). This might appear a contradictory finding to the present finding that Fb‐Skn expressed *Bdnf* more than nerve‐derived Fb. But Fb‐Skn are also reported to express Bdnf (Palazzo et al., 2012), and Bdnf is involved with fibrosis in various organs (Hang et al., 2021), supporting both findings. Another study reported that Fb accumulating at injury site 5 days after PNI expressed EphB2 to sort SCs for forming bridging tissue. But this study did not focus on the origin of the EphB2‐expressing Fb (Parrinello et al., 2010). In another study, Fb and SCs were cultured from postmortem human peripheral nerves, purified by magnetic labelling with antibodies against p75, and subjected to transcriptome analysis (Peng et al., 2020). Although it is not clear that these Fb included Fb‐Epn, this study demonstrated that Fb expressed genes of neurotrophic factors and adhesion molecules for axonal growth and pathfinding, supporting the view that Fb can promote neurite outgrowth and secrete growth factors and extracellular matrix proteins to stimulate SC migration (Dreesmann et al., 2009; Van Neerven et al., 2013; Zhang et al., 2016).

Fibroblasts have been explored as a source of cell therapy, because of their ease of preparation and expansion (Takahashi et al., 2007; Wong et al., 2007). For example, Fb genetically modified to secrete growth factors were transplanted into the lesion site of spinal cord injury to enhance axon regeneration (Krupka et al., 2017; Park et al., 2013; Zhou et al., 2019). For PNI, a nerve defect was reconstructed with three‐dimensional nerve conduits fabricated with Fb (Takeuchi et al., 2020; Zhang et al., 2018). Although these studies used Fb‐Skn, based on the present findings, nerve‐derived Fb might have more potential for use as a cell therapy for PNI. Future studies will be required to determine what types of Fb are optimal for specific purposes as a source of cell therapy.

## 5 CONCLUSION

Nerve‐derived Fb have a greater neurite‐promoting effect than Fb‐Skn, and Fb‐Epn can promote neurite outgrowth more effectively than Fb‐Par. Although both soluble and cell‐surface factors contribute to their neurite‐promoting effect, the soluble factors appear to function for the in vivo regeneration of axons at the site of injury, because there are abundant nerve‐derived Fb at the site of injury, but their direct contact with axons is lacking. Fb‐Epn and Fb‐Par have distinctly different molecular profiles, including genes that are related to biological functions and genes that encode for soluble factors to promote axons. These findings reveal that Fb are molecularly and functionally different depending on their localization in nerve tissue and suggest the possibility of Fb‐Epn being involved with axon regeneration after PNI more than previously thought.

## AUTHOR CONTRIBUTIONS

Conceptualization: Masato Hara and Ken Kadoya. Methodology: Takeshi Endo and Ken Kadoya. Investigation: Masato Hara. Funding acquisition: Takeshi Endo and Ken Kadoya. Project administration: Ken Kadoya. Supervision: Norimasa Iwasaki. Writing: Masato Hara, Takeshi Endo and Ken Kadoya. All authors approved the final version of the manuscript and agree to be accountable for all aspects of the work in ensuring that questions related to the accuracy or integrity of any part of the work are appropriately investigated and resolved. All persons designated as authors qualify for authorship, and all those who qualify for authorship are listed.

## CONFLICT OF INTEREST

None declared.

## Supporting information

Statistical Summary Document

## Data Availability

The datasets generated during and/or analysed during the present study are available from the corresponding author on reasonable request. Sequence data that support the findings of this study were deposited in Gene Expression Omnibus under the accession code GSE211427.
